# {Ni_4_O_4_} Cluster Complex to Enhance the Reductive Photocurrent Response on Silicon Nanowire Photocathodes

**DOI:** 10.3390/nano7020033

**Published:** 2017-02-06

**Authors:** Yatin J. Mange, Soundarrajan Chandrasekaran, Nathan Hollingsworth, Nicolas H. Voelcker, Ivan P. Parkin, Thomas Nann, Thomas J. Macdonald

**Affiliations:** 1 Future Industries Institute, University of South Australia, Mawson Lakes, 5095 SA, Australia; yatinmange@yahoo.com (Y.J.M.); soundarbiotech@gmail.com (S.C.); nico.voelcker@unisa.edu.au (N.H.V.); 2 Department of Chemistry, University College London, London WC1H 0AJ, UK; n.hollingsworth@ucl.ac.uk (N.H.); i.p.parkin@ucl.ac.uk (I.P.P.); 3 MacDiarmid Institute for Advanced Materials and Nanotechnology, School of Chemical and Physical Sciences, Victoria University of Wellington, Wellington 6140, New Zealand; thomas.nann@vuw.ac.nz

**Keywords:** photocathode, nickel oxide, nanowires, water splitting

## Abstract

Metal organic {Ni_4_O_4_} clusters, known oxidation catalysts, have been shown to provide a valuable route in increasing the photocurrent response on silicon nanowire (SiNW) photocathodes. {Ni_4_O_4_} clusters have been paired with SiNWs to form a new photocathode composite for water splitting. Under AM1.5 conditions, the combination of {Ni_4_O_4_} clusters with SiNWs gave a current density of −16 mA/cm^2^, which corresponds to an increase in current density of 60% when compared to bare SiNWs. The composite electrode was fully characterised and shown to be an efficient and stable photocathode for water splitting.

## 1. Introduction

The depletion of fossil fuels and the effects of global warming is a major concern for the world’s future energy requirements. Using inorganic semiconductors to perform water electrolysis under solar radiation has been shown to produce clean, sustainable and renewable fuels such as hydrogen (H_2_) [1]. As an alternative to fossil fuels, H_2_ will play an important role for the future because it is storable, transportable, can be converted to hydrocarbon fuels using the Fischer-Tropsch or Sabatier process and can be converted into electrical energy using fuel cells [2]. Following the work by Fujishima and Honda [1], there has been enormous interest in the field of semiconductor materials for photocatalysis and photoelectrolysis, specifically on the mechanisms that are involved in photoelectrochemical (PEC) water splitting [3,4,5,6,7,8]. The photocatalytic approach for water splitting can be divided into two types. The first involves the use of single visible-light-responsive photocatalysts with sufficient potential to split water into H_2_ and oxygen (O_2_) [9]. The second is via the two-step excitation mechanism using two different photocatalysts, analogous to photosynthesis carried out by plants [10]. In these systems, devices are typically built upon an *n*-type semiconducting photoanode and *p*-type photocathode. Recent advances in photocathodes such as Si [11], Cu_2_O [12], CuO [13,14], and GaInP_2_ [15] have resulted in solar-to-fuel efficiencies are becoming comparable to the much studied photoanodes such as TiO_2_ [16]. This leads to photocathodes being replaced by expensive platinum group metal catalysts. As an alternative to platinum, our previous reports have studied alternative photocathode materials based on nickel oxide (NiO) [17], silicon [18] and carbon nanotubes [19].

Silicon is still the most popular inorganic photoelectrode because of its low band gap, abundance and broad absorption of the solar spectrum. Nanostructured forms of silicon such as silicon nanowires (SiNWs) [18], porous silicon (p-Si) [20], and flat silicon [11] have shown their potential as photocathodes for renewable energy generation [21]. In addition, the tuneable band gap and antireflective properties are some of the features which make SiNWs suitable for water splitting [22]. Recently, Liu et al., described the mechanism for photocatalytic H_2_ production on SiNWs and suggest it is not true water splitting [23]. This has led researchers to combine silicon with molecular catalysts to lower the activation energy and improve the efficiency of solar water splitting [24]. Furthermore, 1-dimensional (1D) structures such as nanowires can offer better charge transport by providing a more direct pathway for charge collection/transfer [25,26]. They have also been shown to reduce the interconnected grain boundaries commonly associated with particulate based photoelectrodes [27].

Recently, materials such as nanocrystals (NCs) have been used as photosensitisers, which aid in maximising the visible light absorption when paired with a silicon semiconductor [28]. An alternative to NCs is to incorporate molecular catalysts made from low-cost, earth abundant elements, which contain well-defined structures. Examples of molecular catalysts that fit this criterion are typically based on first row transition metals iron, copper, cobalt and nickel. As one of earth’s most abundant elements, materials made from nickel have been estimated to be one of the most significant catalysts due to their water oxidation potential.

In this work, we propose the use of a {Ni_4_O_4_} molecular cluster as a reduction co-catalyst with SiNWs to produce a novel photocathode for water splitting. The introduction of {Ni_4_O_4_} molecular clusters significantly increased the reductive photocurrent response of SiNWs compared to bare SiNWs.

## 2. Results and Discussion

**Figure 1:**
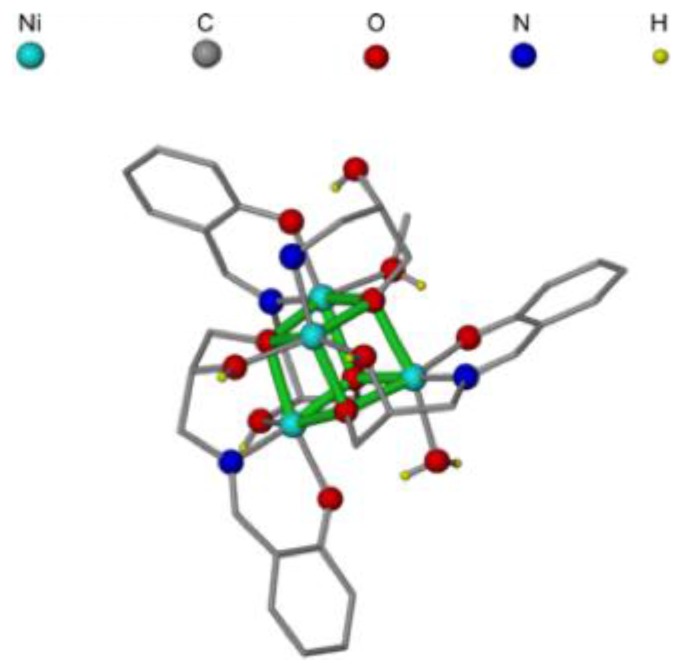
Structure of **1** taken from the Cambridge Crystallographic Data Centre (CCDC). Solvate molecules, anion and all H atoms bonded to C omitted for clarity. Central {Ni_4_O_4_} cubane unit bonds highlighted in green.

The tetra-nuclear {Ni_4_O_4_} cubane cluster complex, [Ni_4_(HL1)_3_(HL2)(H_2_O)(CH_3_OH)][CH_3_COO]·2CH_3_OH·CH_3_CN (**1**) (Figure 1) where (H_3_L1=3-(2-hydroxybenzylideneamino)propane-1,2-diol; H_2_L2=3-aminoprppane-1,2-diol) has been prepared by the reported one-pot reaction of nickel(II) acetate, salicylaldehyde and 3-aminopropane-1,2-diol (see Materials and Methods) [29]. **1** has been chosen as the {Ni_4_O_4_} cluster employed in this study due to its ease of synthesis and its distinctive structure, whereby each nickel centre is in a unique environment. A {Ni_4_O_4_} bio-mimic with each nickel in a differing environment may offer a more accurate mimic to the natural oxygen centre in photo system II, whereby the manganese metal centres are all in different environments owing to their varying organic substituents.

Herein, we paired {Ni_4_O_4_} clusters with SiNW photocathodes forming a new *p*-type photoelectrode for water electrolysis. SiNW photoelectrodes were prepared by metal assisted chemical etching (MACE) and the {Ni_4_O_4_} clusters were loaded into the photocathodes by drop cast technique. The MACE technique helped in the formation of non-ordered SiNW arrays. SiNW array traps the incoming light and helps in the improved solar to current efficiency. Addition of the {Ni_4_O_4_} clusters gave a significant increase in photocurrent response for SiNW photocathodes. Information on the fabrication of SiNWs, {Ni_4_O_4_} clusters and electrode fabrication is mentioned in Materials and Methods.

**Figure 2:**
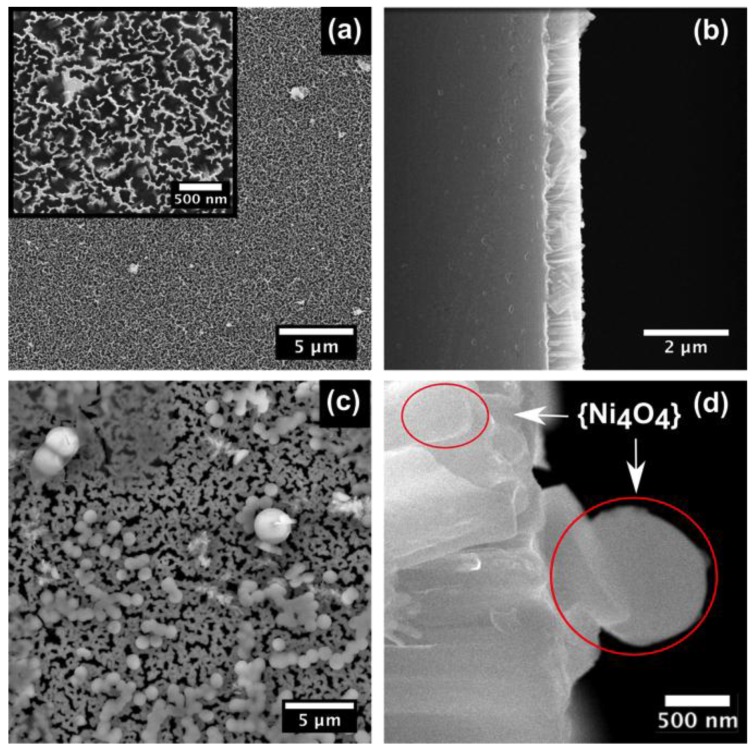
SEM images (**a**,**b**) top and cross section view of bare SiNWs. Inset in Figure 1**a** represents islands of SiNWs; (**c**,**d**) top and cross section view of SiNWs coated with {Ni_4_O_4_} clusters.

The network of NWs can be seen in the scanning electron microscopy (SEM) image in Figure 2. Figure 2a,b shows the top view and cross sectional view of bare SiNWs. The inset in Figure 2a represents the islands of SiNWs, which are distributed across the surface of the substrate. The cross section in Figure 2b represents the bare SiNWs, which were measured to be 1 µm in height using Image J software. Figure 2c,d shows the top and cross sectional view of the SiNWs coated with {Ni_4_O_4_} clusters. Figure 2c shows the SiNWs after drop casting {Ni_4_O_4_} clusters on the surface. The {Ni_4_O_4_} clusters were non-homogeneously distributed across the NW surface. The cross section of the SiNWs + {Ni_4_O_4_} clusters is shown in Figure 2d which shows the clusters of varying sizes (up to 1 µm) on the SiNWs. Additional SEM images can be found in the electronic supporting information (ESI) (Figure S1). The presence of {Ni_4_O_4_} clusters on the SiNWs was confirmed by means of X-ray photoelectron spectroscopy (XPS) and energy dispersive X-ray spectroscopy (EDS). Confirmation of the nickel 2p spectra for the {Ni_4_O_4_} clusters can be found in the ESI (Figure S2). EDS spectra were scanned over the cross sections and the red circle insets indicated the presence of the {Ni_4_O_4_} clusters. The EDS spectra of bare SiNWs and SiNWs with {Ni_4_O_4_} clusters is shown in the ESI (Figure S3a,b).

**Figure 3:**
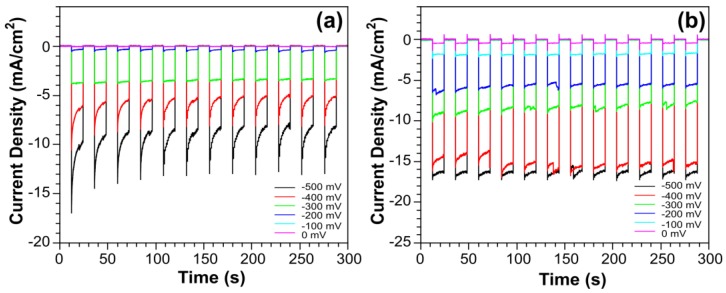
Photocurrent measurements showing current density as a function of time for (**a**) bare SiNWs and (**b**) SiNWs + {Ni_4_O_4_} clusters.

Figure 3a shows the current density as a function of time for the SiNW photocathodes. All current density measurements were obtained by ramping the bias potential between 100 and 500 mV for 5 min under air mass (AM) 1.5 conditions. Bare SiNW photocathodes gave a maximum current density of −10 mA/cm^2^, which was measured with an applied bias potential of −500 mV. Figure 3b shows the current density for the SiNW photocathodes containing the {Ni_4_O_4_} catalyst. The addition of the {Ni_4_O_4_} catalyst gave a maximum current density of −16 mA/cm^2^, which corresponds to an improvement in current density of 60%. This is the first example of {Ni_4_O_4_} acting as a reduction catalyst as opposed to an oxidation catalyst [30]. The current densities were measured by ramping the bias potential in 100 mV increments in 0.1 M H_2_SO_4_ electrolyte vs. Ag/AgCl.

**Figure 4:**
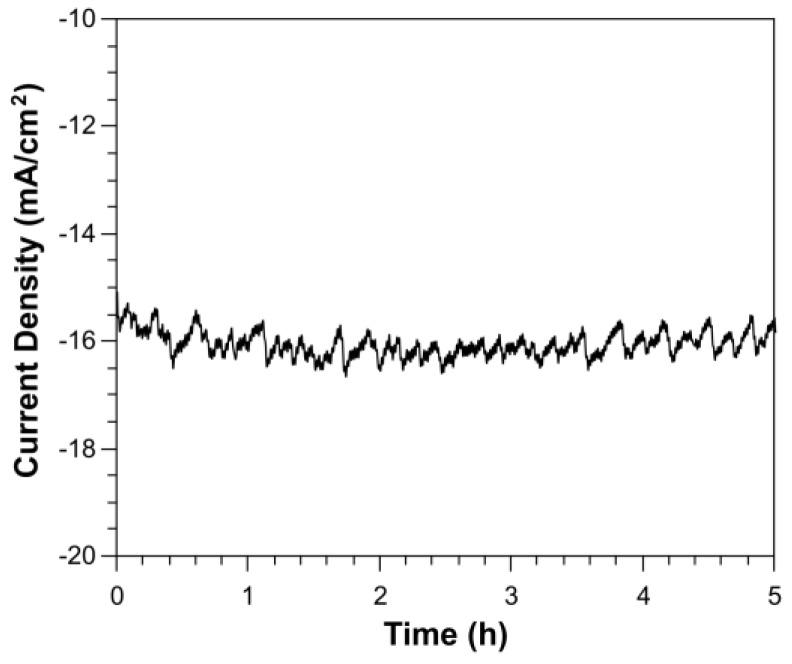
Current density measurement of SiNWs + {Ni_4_O_4_} clusters catalyst in 0.1 M H_2_SO_4_ at a bias potential of −500 mV over 5 h.

A time course study showed that the current density is stable under illumination for at least 5 h (Figure 4). Passivation techniques such as electrografting, thermal hydrocarbonisation and hydrosilylation of SiNW photocathodes for improving stabilities are currently being explored. H_2_ was measured by taking 500 µL aliquots from the headspace of the photoelectrochemical cell after 1 h [31]. The evolution of H_2_ was quantified by gas chromatography to be approximately 279 µLmol/h.

## 3. Materials and Methods

Nickel (II) acetate-tetrahydrate (Ni(OAc)_2_·4H_2_O), salicylaldehyde, 3-aminopropane-1,2-diol, sodium hydroxide, methanol and acetonitrile were all purchased from Sigma-Aldrich (Castle Hill, NSW, Australia) and used as such without further purification.

SiNWs were fabricated from *p*-type silicon wafers (Czochralski, Silicon Quest Intl. Ltd., San Jose, CA, USA) with resistivity of 10–20 mΩ·cm, orientation (100). Hydrofluoric acid (HF, 48%) was purchased from Scharlau Chemie (Chem-Supply Pty. Ltd., Gillman, SA, Australia). Silver nitrate (AgNO_3_) and hydrogen peroxide (H_2_O_2_, 30%) were purchased from Merck (Bayswater, VIC, Australia).

### 3.1. Synthesis of {Ni_4_O_4_} Clusters

{Ni_4_O_4_} clusters, **1**, were synthesised according to the previously reported literature method [29]. In a typical experiment, Ni(OAc)_2_·4H_2_O (4 mmol), salicylaldehyde (4 mmol), 3-aminopropane-1,2-diol (5 mmol) and sodium hydroxide (8 mmol) was mixed with methanol (50 mL) in a reaction flask. The resulting green mixture was stirred at room temperature for 40 h. Following this, acetonitrile (20 mL) was added and the resulting green solution was allowed to evaporate at room temperature resulting in green crystals of {Ni_4_O_4_} clusters. The green crystals were analysed by Attenuated total reflection spectroscopy (ATIR) on a Bruker (Bruker Optics, Billerica, MA, USA) Alpha Platinum-ATR showing a good match to **1**. IR found (ATR)/[IR previously reported [29] (KBr)]: *υ*_max_/cm^−1^ = 3410 (s)/[3448 (s)], 2917 (m)/[2917 (m)], 2846 (m)/[2850 (m)], 1638 (s)/[1627 (s)], 1595 (m)/[1596 (m)], 1542 (m)/[1541 (m)], 1472 (m)/[1472 (m)], 1308 (m)/[1310 (m)], 1104 (s)/[1104 (s)], 1034 (s)/[1039 (s)], 755 (m)/[761 (m)], 591 (m)/[598 (m)].

Mass spectrometry was performed on a Waters LCT Premier XE ESI Q-TOF mass spectrometer (Waters, Milford, MA, USA) isotope patterns were typical for a {Ni_4_O_4_} molecular ion. m/z (ES+) 1040.0 (M^+^-[CH_3_COO]-H_2_O), 1007.0 (M-[CH_3_COO]-CH_3_OH-H_2_O), 935.0 (M-[CH_3_OO]-2CH_3_OH·CH_3_CN-H_2_O). UV-Vis of the {Ni_4_O_4_} clusters can be found in the supporting information (Figure S4).

### 3.2. Fabrication of SiNWs

A *p*-type silicon wafer with resistivity of 10–20 mΩ·cm was cleaned by ultrasonication using acetone, ethanol and deionised water for 5 min, respectively. The wafers were then cut into 1 × 1 cm^2^ pieces and placed in 1:1 HF and ethanol solution in order to remove the native oxide layers. The unpolished side of the wafers were masked using a sticky tape to avoid etching of the surface. Firstly, the wafers were dipped in 4.8 M HF and 0.02 M AgNO_3_ solution for 30 s in order to deposit silver (Ag) on the polished side. The wafers were then immediately dipped in the etching solution of 4.8 M HF and 0.1 M H_2_O_2_ for 2 min. The etched wafers were then rinsed with deionised water and the sticky tape was removed from the unpolished side. Finally, the etched wafers were dipped in conc. nitric acid for 20 min to remove Ag coating, then washed with deionised water and transferred into an Argon purged glove box.

### 3.3. Electrode Fabrication

Initially the 1 × 1 cm^2^ SiNW piece was dipped in 1:1 HF and ethanol solution for 30 s to remove the native oxide layer. It was then dried in a stream of N_2_ gas and quickly transferred to an argon purged glove box. {Ni_4_O_4_} (1 mg in 1 mL methanol) clusters (**1**) were deposited on SiNWs by “drop casting” procedure inside the glove box. Few drops of {Ni_4_O_4_} in methanol were placed on the SiNW sample and then allowed to dry. This was repeated five times. A back contact to the SiNWs photocathode (unetched surface) was obtained by using In-Ga eutectic. A copper plate was used as a rigid electrical contact.

## 4. Conclusions

In conclusion, we have developed a new SiNW photocathode for water splitting that achieves a 60% improvement in current density when compared to bare SiNWs. The combination of {Ni_4_O_4_} clusters with SiNWs gave a current density of −16 mA/cm^2^ in contrast to −10 mA/cm^2^ for bare SiNW photocathodes. While {Ni_4_O_4_} clusters are known oxidation catalysts [32], here we show them to also be valuable in increasing the reductive photocurrent response on SiNW photocathodes for water splitting. The resultant photocathode is an example of an efficient electrode made from abundant materials, which is capable of splitting water.

## Supplementary Materials

The following are available online at http://www.mdpi.com/2079-4991/7/2/33/s1.

## References

[1] Fujishima A., Honda K. (1972). Electrochemical Photolysis of Water at a Semiconductor Electrode. Nature.

[2] Contestabile M., Offer G.J., Slade R., Jaeger F., Thoennes M. (2011). Battery electric vehicles, hydrogen fuel cells and biofuels. Which will be the winner?. Energy Environ. Sci..

[3] Balzani V., Moggi L., Manfrin M.F., Bolletta F., Gleria M. (1975). Solar Energy Conversion by Water Photodissociation Transition metal complexes can provide low-energy cyclic systems for catalytic photodissociation of water. Science.

[4] Bolton J.R., Strickler S.J., Connolly J.S. (1985). Limiting and realizable efficiencies of solar photolysis of water. Nature.

[5] Tachibana Y., Vayssieres L., Durrant J.R. (2012). Artificial photosynthesis for solar water-splitting. Nat. Photonics.

[6] Kawasaki S., Takahashi R., Yamamoto T., Kobayashi M., Kumigashira H., Yoshinobu J., Komori F., Kudo A., Lippmaa M. (2016). Photoelectrochemical water splitting enhanced by self-assembled metal nanopillars embedded in an oxide semiconductor photoelectrode. Nat. Commun..

[7] Sarnowska M., Bienkowski K., Barczuk P.J., Solarska R., Augustynski J. (2016). Highly Efficient and Stable Solar Water Splitting at (Na)WO_3_ Photoanodes in Acidic Electrolyte Assisted by Non-Noble Metal Oxygen Evolution Catalyst. Adv. Energy Mater..

[8] Touge T., Nara H., Fujiwhara M., Kayaki Y., Ikariya T. (2016). Efficient Access to Chiral Benzhydrols via Asymmetric Transfer Hydrogenation of Unsymmetrical Benzophenones with Bifunctional Oxo-Tethered Ruthenium Catalysts. J. Am. Chem. Soc..

[9] Lee Y., Terashima H., Shimodaira Y., Teramura K., Hara M., Kobayashi H., Domen K., Yashima M. (2007). Zinc Germanium Oxynitride as a Photocatalyst for Overall Water Splitting under Visible Light. J. Phys. Chem. C.

[10] Abe R., Shinmei K., Hara K., Ohtani B. (2009). Robust dye-sensitized overall water splitting system with two-step photoexcitation of coumarin dyes and metal oxide semiconductors. Chem. Commun..

[11] Ji L., McDaniel M.D., Wang S., Posadas A.B., Li X., Huang H., Lee J.C., Demkov A.A., Bard A.J., Ekerdt J.G. (2015). A silicon-based photocathode for water reduction with an epitaxial SrTiO_3_ protection layer and a nanostructured catalyst. Nat. Nanotechnol..

[12] Luo J., Steier L., Son M.-K., Schreier M., Mayer M.T., Gratzel M. (2016). Cu_2_O Nanowire Photocathodes for Efficient and Durable Solar Water Splitting. Nano Lett..

[13] Masudy-Panah S., Moakhar R.S., Chua C.S., Kushwaha A., Wong T.I., Dalapati G.K. (2016). Rapid thermal annealing assisted stability and efficiency enhancement in a sputter deposited CuO photocathode. RSC Adv..

[14] Masudy-Panah S., Siavash Moakhar R., Chua C.S., Tan H.R., Wong T.I., Chi D., Dalapati G.K. (2016). Nanocrystal Engineering of Sputter-Grown CuO Photocathode for Visible-Light-Driven Electrochemical Water Splitting. ACS Appl. Mater. Interfaces.

[15] Gu J., Yan Y., Young J.L., Steirer K.X., Neale N.R., Turner J.A. (2016). Water reduction by a p-GaInP_2_ photoelectrode stabilized by an amorphous TiO_2_ coating and a molecular cobalt catalyst. Nat. Mater..

[16] Khan S.U.M., Al-Shahry M., Ingler W.B. (2002). Efficient Photochemical Water Splitting by a Chemically Modified n-TiO_2_. Science.

[17] Macdonald T.J., Mange Y.J., Dewi M.R., Islam H.U., Parkin I.P., Skinner W.M., Nann T. (2015). CuInS_2_/ZnS nanocrystals as sensitisers for NiO photocathodes. J. Mater. Chem. A.

[18] Chandrasekaran S., Macdonald T.J., Mange Y.J., Voelcker N.H., Nann T. (2014). A quantum dot sensitized catalytic porous silicon photocathode. J. Mater. Chem. A.

[19] Macdonald T.J., Tune D.D., Dewi M.R., Bear J.C., McNaughter P.D., Mayes A.G., Skinner W.M., Parkin I.P., Shapter J.G., Nann T. (2016). SWCNT photocathodes sensitised with InP/ZnS core–shell nanocrystals. J. Mater. Chem. C.

[20] Chandrasekaran S., McInnes S.J.P., Macdonald T.J., Nann T., Voelcker N.H. (2015). Porous silicon nanoparticles as a nanophotocathode for photoelectrochemical water splitting. RSC Adv..

[21] Chandrasekaran S., Nann T., Voelcker N.H. (2015). Nanostructured silicon photoelectrodes for solar water electrolysis. Nano Energy.

[22] Wu X., Kulkarni J.S., Collins G., Petkov N., Almecija D., Boland J.J., Erts D., Holmes J.D. (2008). Synthesis and Electrical and Mechanical Properties of Silicon and Germanium Nanowires. Chem. Mater..

[23] Liu D., Li L., Gao Y., Wang C., Jiang J., Xiong Y. (2015). The Nature of Photocatalytic “Water Splitting” on Silicon Nanowires. Angew. Chem. Int. Ed..

[24] Hou Y., Abrams B.L., Vesborg P.C.K., Bjorketun M.E., Herbst K., Bech L., Setti A.M., Damsgaard C.D., Pedersen T., Hansen O. (2011). Bioinspired molecular co-catalysts bonded to a silicon photocathode for solar hydrogen evolution. Nat. Mater..

[25] Macdonald T.J., Tune D.D., Dewi M.R., Gibson C.T., Shapter J.G., Nann T. (2015). A TiO_2_ Nanofiber–Carbon Nanotube-Composite Photoanode for Improved Efficiency in Dye-Sensitized Solar Cells. ChemSusChem.

[26] Tran D.P., Macdonald T.J., Wolfrum B., Stockmann R., Nann T., Offenhausser A., Thierry B. (2014). Photoresponsive properties of ultrathin silicon nanowires. Appl. Phys. Lett..

[27] Mor G.K., Shankar K., Paulose M., Varghese O.K., Grimes C.A. (2005). Enhanced Photocleavage of Water Using Titania Nanotube Arrays. Nano Lett..

[28] Nann T., Ibrahim S.K., Woi P.-M., Xu S., Ziegler J., Pickett C.J. (2010). Water Splitting by Visible Light: A Nanophotocathode for Hydrogen Production. Angew. Chem. Int. Ed..

[29] Zhang S.-Y., Chen W.-Q., Hu B., Chen Y.-M., Li W., Li Y. (2012). A unique tetranuclear cubane-like {Ni_4_O_4_} complex supported by hydroxyl-rich ligands: Synthesis, crystal structure and magnetic property. Inorg. Chem. Commun..

[30] Baktash E., Littlewood P., Pfrommer J., Schomacker R., Driess M., Thomas A. (2015). Controlled Formation of Nickel Oxide Nanoparticles on Mesoporous Silica using Molecular {Ni_4_O_4_} Clusters as Precursors: Enhanced Catalytic Performance for Dry Reforming of Methane. ChemCatChem.

[31] Chandrasekaran S., Macdonald T.J., Gerson A.R., Nann T., Voelcker N.H. (2015). Boron-Doped Silicon Diatom Frustules as a Photocathode for Water Splitting. ACS Appl. Mater. Interfaces.

[32] Han X.-B., Li Y.-G., Zhang Z.-M., Tan H.-Q., Lu Y., Wang E.-B. (2015). Polyoxometalate-Based Nickel Clusters as Visible Light-Driven Water Oxidation Catalysts. J. Am. Chem. Soc..

