# Supplementary material for: {Ni4O4} Cluster Complex to Enhance the Reductive Photocurrent Response on Silicon Nanowire Photocathodes
Source: Nanomaterials (Basel). 2017 Feb 6;7(2):33. doi: 10.3390/nano7020033 (PMC5333018; doi:10.3390/nano7020033)
Supplement: Supplementary file 1 [file nanomaterials-07-00033-s001.pdf]

Supplementary Materials: $\{Ni_4O_4\}$ Cluster Complex to Enhance the Reductive Photocurrent Response on Silicon Nanowire Photocathodes

Yatin J. Mange, Soundarrajan Chandrasekaran, Nathan Hollingsworth, Nicolas H. Voelcker, Ivan P. Parkin, Thomas Nann and Thomas J. Macdonald

Photocurrent and GC measurements:

Photocurrent experiments were performed using an Abet solar simulator (air mass 1.5–1 sun) and calibrated against a silicon solar cell (New-Spec). Electrochemical measurements were carried out using PG 310 potentiostat from HEKA Electronics (Lambrecht/Pfalz, Germany). Electrolysis experiments were performed using a sealed three-electrode Teflon PEC cell consisting of a platinum counter electrode, Ag/AgCl 3M KCl reference electrode and the working electrode (SiNWs with $\{Ni_4O_4\}$ clusters). The working electrode was illuminated with a light intensity of 100 mW/cm^2 under air mass 1.5 conditions with short 12 s dark and 12 s light cycle to measure the photocurrents for 5 min. The potential between the working and reference electrodes was adjusted between 0 and −500 mV in 100 mV steps. The 5-h photocurrent run was measured using a light cycle at a bias voltage of −500 mV with respect to the open circuit potentials (OCPs). In order to measure H_2, the sample gas in the headspace (500 μL) above the electrolyte was sampled after 1 h and analysed using a SRI 310C series GC with a thermal conductivity detector and a column held at 70 °C using N_2 as the carrier gas.

Surface Characterisation:

Scanning electron microscopy (SEM) images were obtained using an FEI Quanta 450 environmental scanning electron microscope (Hillsboro, OR, USA). X-ray photoelectron spectroscopy (XPS) measurements were recorded on a Thermo Scientific K-alpha spectrometer (Staffordshire Technology Park, Stafford, UK) at University College London.

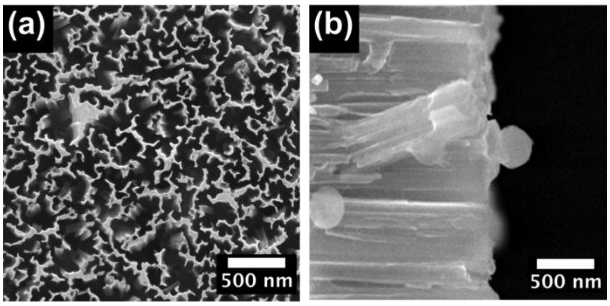

Figure S1. (**a**) Top down SEM for SiNW photocathodes (bare); (**b**) shows a cross section SEM of the SiNWs with $\{Ni_4O_4\}$ co-catalyst.

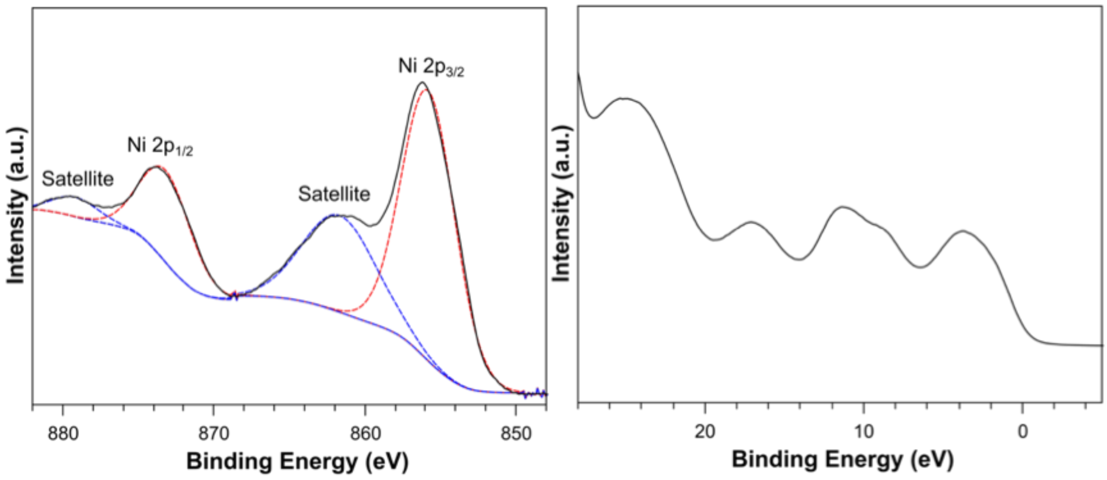

Figure S2. Ni 2p XPS spectra of {Ni_4O_4} clusters (**left**), valence spectra for Ni 2 p (**right**).

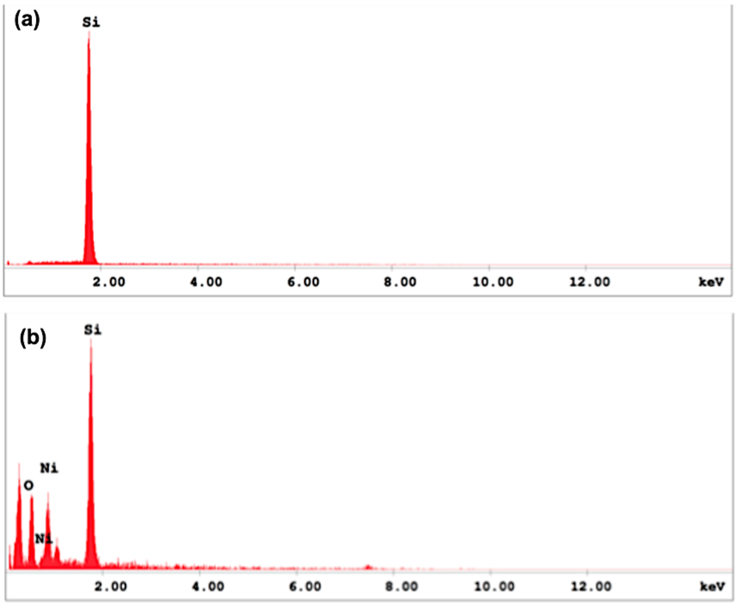

Figure S3. EDS spectra of (**a**) bare SiNWs and (**b**) SiNWs + {Ni_4O_4} clusters.

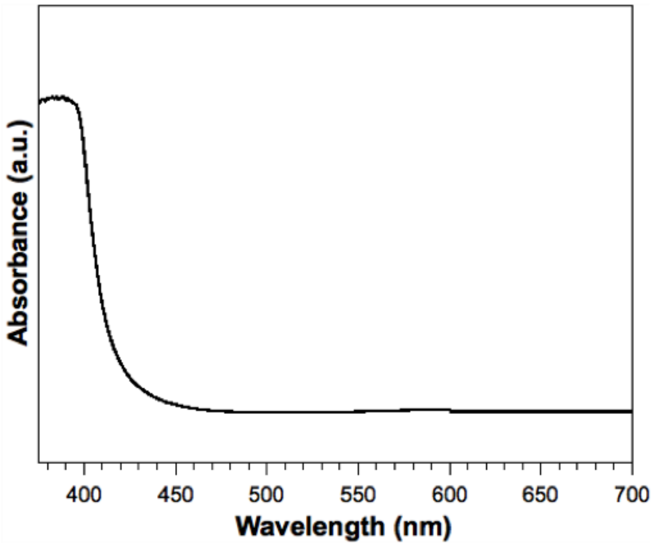

Figure S4. UV-visible spectroscopy of {Ni_4O_4} clusters.
